# Protecting healthcare workers from SARS-CoV-2 and other infections

**DOI:** 10.1017/S0950268820002198

**Published:** 2020-09-21

**Authors:** Mengding Chen, Xin Wei, Zhengguang Wang

**Affiliations:** 1 First Affiliated Hospital of Anhui Medical University, Hefei 230000, China; 2 Department of General Surgery for Cadres, First Affiliated Hospital of Anhui Medical University, Hefei 230000, China

**Keywords:** COVID-19, healthcare workers, SARS-CoV-2

## Abstract

Coronavirus disease 2019 (COVID-19) has had a tremendous impact in China and abroad since its onset in December 2019 and poses a major threat to human health. Healthcare workers (HCWs) are at the forefront of the response to outbreaks. This study reviewed literature data and found that HCWs were at high risk of infection during the COVID-19 pandemic, especially at the early stage of the epidemic, and many factors greatly affected their occupational safety. Although SARS-CoV-2 transmission was controlled in China, the Chinese experience can help protect HCWs from COVID-19 and other respiratory diseases.

## Introduction

The first cases of coronavirus disease 2019 (COVID-19), caused by severe acute respiratory syndrome coronavirus 2 (SARS-COV-2), were reported in Wuhan, Hubei province, China, in December 2019 [1, 2], and this disease has had a significant impact on the social and economic development of China and other countries. As of 17 May 2020, approximately 4 525 497 cases were confirmed worldwide, including 84 484 cases in China, 1 870 545 in Europe, 1 966 932 in the Americas and 603 536 in other regions [3, 4]. The estimated case fatality rate of COVID-19 (this rate was used because limited testing capacity prevented determining the actual mortality rate) is 6.8% but varies between countries [4] and is lower than that of severe acute respiratory syndrome (SARS) (9.5%) and Middle East respiratory syndrome (MERS) (34.4%) [5] (Table 1). The National Health Commission of the People's Republic of China reported that, as of 24 February, 3387 healthcare workers (HCWs) had been infected with SARS-CoV-2 in China, and 22 (0.6%) died [6].

**Table 1.** Pathogenicity and transmission characteristics of SARS, MERS and COVID-19

Disease	Case-fatality rate (%)	Proportion of cases among healthcare workers relative to total confirmed cases (%)	Rate of nosocomial transmission (%)
SARS	9.5	21	58
MERS	34.4	18.6	70
COVID-19	4.9^a^	See Table 2	–

a As of 1 April 2020.

**Table 2.** Proportion of healthcare workers diagnosed with SARS-CoV-2 relative to total confirmed cases during the COVID-19 outbreak

Country	Early stage	Late stage
China	29% (40/138)^a^	4.4% (3387/77 262)^b^
Italy	20% (350/1350)^c^	10% (1225/12 252)^d^

a From 1 January to 28 January 2020, in Zhongnan Hospital of Wuhan University, Wuhan, China.

b As of 24 February 2020.

c As of 12 March 2020.

d As of 5 April 2020.

The basic reproduction number (R0) is a common measure of transmissibility and is defined as the average number of people infected by one individual over the course of illness. R0 values lower than 1 indicate that transmission is interrupted, R0 values higher than 1 indicate that transmission is sustained [7, 8]. R0 was >2 and <1 at the early and late stage of the 2003 SARS outbreak, respectively [9]. R0 ranged from 2.2 to 3.9 during the COVID-19 epidemic in China, depending on the disease transmission model used and testing capacity [8, 10, 11], and tended to decrease as the epidemic was controlled and effective policies were implemented.

COVID-19 is a major threat to human health, and HCWs in affected countries can benefit from the Chinese experience in managing this pandemic. The purpose of this study is to discuss the Chinese experience to help protect HCWs from viral outbreaks.

## Occupational exposure of HCWs to coronaviruses

SARS-CoV-2 is the seventh coronavirus strain known to infect humans since the first human coronavirus was identified in the 1960s [12]. Four strains – NL-63, 229E, HKU1 and OC43 – cause mild illness, whereas three strains – MERS-CoV, SARS-CoV and SARS-CoV-2 – may cause severe disease [13]. MERS-CoV and SARS-CoV were a public health threat in some countries, whereas SARS-CoV-2 has had a significant health and economic impact worldwide. The number of nosocomial cases during the 2003 SARS and 2012 MERS outbreaks corresponded to 58% and 70% of all confirmed cases, respectively [5] (Table 1).

The COVID-19 pandemic put global public health authorities (PHAs) on high alert. HCWs are at the forefront of the response to communicable diseases and, therefore, have a higher risk of viral exposure. Among all detected MERS-CoV and SARS-CoV infection cases, 18.6% and 21% occurred in HCWs, respectively [14, 15]. In addition, the prevalence of COVID-19 was higher among HCWs than in the general population in several countries [16].

SARS-CoV-2 infections in HCWs accounted for 29% (40/138) of all cases treated in Zhongnan Hospital of Wuhan University in Wuhan, China, from 1 January to 28 January 2020^1^ [17] and 4.4% (3387/77 262) of all cases reported in China until 24 February 2020 [6, 18]. Similarly, in Italy, SARS-CoV-2 infections in HCWs accounted for approximately 20% (350/1350) of cases notified until 12 March 2020 [19] and 10% of cases (1225/12 252) reported until 5 April 2020 [20] (Table 2), suggesting that transmission to HCWs decreased as the outbreak progressed. The use of personal protective equipment (PPE) may have reduced viral transmission to HCWs in China [21], and improvements in testing capacity have been shown to reduce hospital transmission [22–24].

## Challenges in the early response to COVID-19 in China

**Fig. 1.**
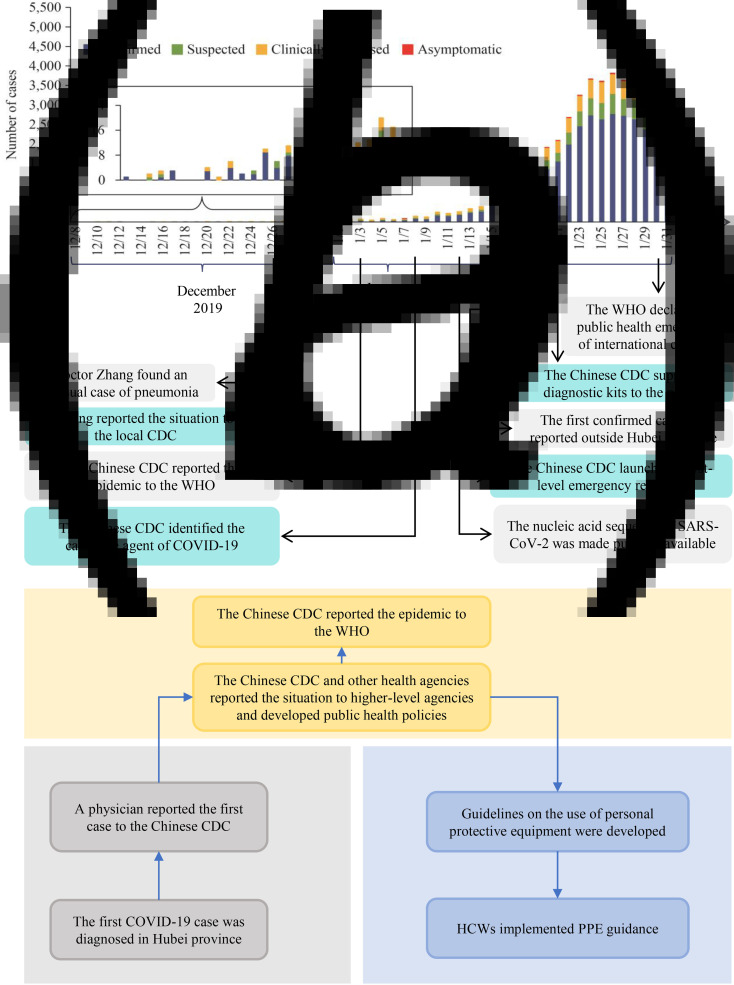
(a) Number of cases of COVID-19 diagnosed between 8 December 2019, and 31 January 2020, in China (adapted from Wu *et al*. [1]). (b) Response of healthcare workers in the early stage of the COVID-19 outbreak in China.

HCWs actively participated in controlling the outbreak during the period from the diagnosis of the first case in Wuhan to public acknowledgement of the situation. The timeline of the COVID-19 outbreak in China is shown in Figure 1a (adapted from Wu *et al*. [1]) [11, 25, 26]. Despite the clinical similarities between SARS and COVID-19, the response of Chinese PHAs to the latter was better regarding the number of confirmed cases before the day the WHO was notified (300 and 27, respectively), number of deaths (five and zero, respectively) [25] and the period from WHO notification to the identification of the etiological agent (2 months and 1 week, respectively) [25]. The period between the notification of the first cases to the Chinese CDC and the identification of the causative pathogen was 12 days, and the period from pathogen identification to the declaration of public health emergency was 22 days. Although great progress has been made, there is room for improving the prevention and control of SARS-CoV-2 transmission among HCWs.

The emergency response period can be divided into three stages (Fig. 1b): (1) from clinical detection to the reporting of the first case to PHAs by HCWs; (2) from the notification of PHAs to decision-making; (3) from decision-making to the provision of new guidelines to HCWs. Four challenges negatively impacted the emergency response:

- Disease aetiology was unknown at the beginning of the epidemic, and new cases were reported to PHAs by HCWs
- The lack of clinical and epidemiological data delayed decision-making
- The lack of PPE and low testing capacity limited the prevention and control of COVID-19 by HCWs
- Delays in communication hindered the ability of HCWs to adopt adequate self-protection measures

## Suggestions and expectations

These challenges can be overcome using the following approaches.

### Improvement of the emergency response

Shortening the period between the diagnosis of the first case and reporting the situation to the local CDC is paramount. However, in the face of unknown diseases, relying on the experience of HCWs to treat patients and report unusual cases is not enough, and adequate surveillance and early warning systems are necessary to interrupt the spread of respiratory viruses. Other factors that reduce the effectiveness of the emergency response are the limited focus on disease prevention and control, insufficient understanding of the epidemic situation by decision-makers and lack of public awareness of the situation [27]. Addressing these problems is crucial to prevent and control new cases and protect HCWs in a timely manner. These goals can be achieved in China by improving disease surveillance systems and public health, increasing the number of public health workers in hospitals and government agencies, and increasing public health knowledge through the media and other communication channels.

### Collection, analysis and reporting of clinical and epidemiological data

Epidemiological data are essential for accurate diagnosis but are not collected in many hospitals in China. The emergency response can be further strengthened by (1) rigorously collecting epidemiological data through testing, including contact history, especially in emergency and respiratory departments; (2) hiring specialised personnel to analyse epidemiological data, including incidence rates, mortality and the distribution of disease cases, and inform the appropriate authorities about changes in epidemiological patterns. Since SARS, hospitals in China have realised the importance of epidemiological data.

### Use of PPE by HCWs and nucleic acid testing

Many infectious diseases are transmitted by droplets and cause respiratory symptoms and fever. However, many physicians in China did not wear face masks in the hospital setting before the COVID-19 pandemic. In some hospitals, outpatient infrastructure is limited, and ventilation and disinfection are inadequate, which may increase the rate of nosocomial infections, underscoring the need to improve outpatient services to control infections. Furthermore, fever clinics help reduce nosocomial transmission during outbreaks [28] and can be introduced in respiratory and emergency departments for fever screening. The risk of nosocomial infections among HCWs was higher at the initial stage of the COVID-19 epidemic because the availability of PPE was limited at this stage [29–31]. Therefore, the adequate supply of PPE to HCWs and guidelines on its correct use are fundamental to prevent and control the spread of respiratory infectious diseases [32–34].

Studies showed that PCR screening might reduce viral transmission among HCWs [22–24]. In this context, hospitals should test all HCWs and patients routinely whenever possible. China improved the research, development and production of diagnostic reagents during the COVID-19 pandemic and reduced disease transmission despite the limited availability of PPE and low testing capacity at the early stage of the outbreak.

### Improve the communication between PHAs and hospitals

During the COVID-19 epidemic, the communication between PHAs and hospitals was inadequate in China, and the activities of these entities were not coordinated, resulting in underreporting and failure of HCWs to promptly obtain disease information after identifying the first cases, consequently increasing the risk of hospital-acquired infections. However, the National Health Committee stressed the need to establish communication and cooperation between these institutions during outbreaks. In this respect, the following actions can be carried out to overcome these limitations: (1) establishment of new mechanisms of communication, information exchange, and cooperation between PHAs and HCWs at the national level (via the National Health Committee) and local level, including joint training on outbreak management; (2) prompt reporting of new cases to PHAs; (3) establishment of guidelines by PHAs for diagnosis, case reporting, treatment and self-protection. These approaches stimulate medical staff to adopt protective measures and facilitate the timely collection of epidemiological data to accurately assess the epidemic situation and make appropriate decisions.

## Data Availability

The datasets used and/or analysed during the current study are available from the corresponding author on reasonable request.

## Footnotes

1 This rate was estimated using hospital data because of the limited access to public data in China.
